# Panorama of Breakthrough Infection Caused by SARS-CoV-2: A Review

**DOI:** 10.3390/medicina58121733

**Published:** 2022-11-27

**Authors:** Qinglu Fan, Zhihao Nie, Songping Xie

**Affiliations:** Department of Thoracic Surgery, Renmin Hospital of Wuhan University, Wuhan 430060, China

**Keywords:** COVID-19, SARS-CoV-2, variants, Omicron, breakthrough infection

## Abstract

Since the outbreak of the novel coronavirus disease 2019 (COVID-19) in 2019, many countries have successively developed a variety of vaccines against severe acute respiratory syndrome coronavirus 2 (SARS-CoV-2). However, with the continuous spread of SARS-CoV-2, it has evolved several variants; as a result, prevention and control of the pandemic of SARS-CoV-2 has become more important. Among these variants, the Omicron variant has higher transmissibility and immune escape ability and is the main variant causing a large number of COVID-19 breakthrough infection, thus, presenting new challenges to pandemic prevention and control. Hence, we review the biological characteristics of the Omicron variant and discuss the current status and possible mechanism of breakthrough infection caused by the Omicron variant in order to provide insights into the prevention and control of the pandemic of SARS-CoV-2.

## 1. Introduction

From the outbreak of novel coronavirus disease 2019 (COVID-19) in 2019 to 18 October 2022, the number of infections caused by severe acute respiratory syndrome coronavirus 2 (SARS-CoV-2) was 624 million and the number of death was nearly 6.56 million worldwide [1]. Meanwhile, with the spread of SARS-CoV-2, which is susceptible to mutation, including changing the affinity of the virus to host cells, the virus has mutated into five variants of concern (VOCs) [2]: Alpha (B.1.1.7), Beta (B.1.351), Gamma (P.1), Delta (B.1.617.2) and the Omicron variant (B.1.1.529). In order to control the COVID-19 pandemic, various vaccines have been developed and administered [3,4]. However, the number of reported cases with breakthrough infection caused by the Omicron variant is increasing. The Omicron variant, the main variant now, not only has enhanced transmissibility and infectivity but also improved immune escape ability, which results in a large number of SARS-CoV-2 breakthrough infections and poses a huge medical burden on global public health security [5]. Hence, this article mainly discusses the biological characteristics of the Omicron variant, status quo and possible mechanism of breakthrough infection caused by the Omicron variant, as well as some coping measures, which potentially help to combat this virus, in order to better prevent and control the pandemic of SARS-CoV-2.

## 2. Characteristics of the Omicron Variant

Since the Omicron variant was first reported in South Africa and Botswana on 24 November 2021, it has rapidly replaced Delta as the dominant strain worldwide. Compared with other VOCs, Omicron has significantly evolutionary characteristics [5,6,7,8,9,10,11,12], such as: higher infectivity, transmissibility and immune escape ability but reduced pathogenicity. These characteristics have changed the situation of the pandemic and posed a great threat to the global economy, normal life and public health.

### 2.1. Higher Infectivity and Transmissibility

At the end of November 2021, South Africa’s National Institute for Communicable Diseases (NICD) reported that the basic reproduction number (R0) of the Omicron variant in Gauteng Province was vastly higher than that of the Delta variant prevalent in September 2021, indicating that Omicron may be more infectious than Delta [13]. An artificial intelligence model, which had been trained with tens of thousands of experimental data and extensively validated by experimental results on SARS-CoV-2, revealed that Omicron may be over 10 times more infectious than the original virus and approximately 2.8 times as infectious as Delta [12]. That means that Omicron could be the most contagious variant ever. Liu et al. found that the average R0 of the Omicron variant was 9.5 (95% CI: 5.5–24), which was 2.5 times higher than that of Delta [14]. In addition, the UK Health Security Agency reported that the secondary attack rates among contacts of Omicron cases in households (13.6%; 95% CI: 13.1–14.1%) and non-household settings (7.6%; 95% CI: 7.2–8.0%) were higher than those for Delta (household: 10.1%; 95% CI: 10.0–10.2%, non-household: 2.8%; 95% CI: 2.7–2.9%) [6]. Moreover, the transmissibility of Omicron is 8 times higher than the D614G original strain of SARS-CoV-2 [15]. In addition, Omicron is approximately 3.2 times more transmissible than Delta, and the doubling time is proximately three days [6,16]. Of note, the Omicron variant has been shown to result in a higher proportion of asymptomatic infections than other variants, which may be one of the reasons for its rapid spread [17].

### 2.2. Higher Immune Escape Ability but Reduced Pathogenicity

The Omicron variant can escape neutralization by antibodies induced by SARS-CoV-2 vaccines. Studies have confirmed that the activity of neutralizing antibodies in serum after vaccination for Omicron decreased, and the result of a South African real-word study showed that the effectiveness of SARS-CoV-2 vaccines against Omicron decreased to 70% 14 days after vaccination, indicating that Omicron has immune escape ability [18,19]. Moreover, Espenhain et al. found that the proportion of vaccinated or obtaining-booster patients with Omicron and Delta were 83.1% and 53.2%, respectively, based on data from Denmark [11]. Cele et al. found that the activity of neutralizing antibodies induced by the BNT162b2 vaccine against Omicron was approximately 22-fold lower than that of the wild strain [20]. Moreover, studies have confirmed that extensive mutations in the Omicron spike protein destroyed the activity of major potent monoclonal antibodies (mAbs), which leads to a severe reduction in the neutralizing ability of serum after natural infection or vaccination [20,21]. Fortunately, Redd et al. have shown that Omicron did not have many mutations conducive to escaping from T lymphocyte immunity, and SARS-CoV-2-specific CD8+T lymphocytes in recovered patients can still identify Omicron [22]. These studies suggest that the immune escape ability of Omicron from the humoral immunity induced by infection is stronger than that of Delta but has little effect on cellular immunity induced by infection.

Fortunately, Omicron results in milder clinical symptoms than Delta [23], including cough, fatigue, stuffy nose and runny nose, fever, nausea and vomiting, tachypnea and dyspnea, diarrhea, loss of taste or smell, etc., while loss of taste or smell was less common in Omicron [24]. Based on data from Denmark, Espenhain et al. found that the hospitalization rates of patients with Omicron and Delta were 1.2% and 1.5%, ICU occupancy rates were 0.13% and 0.11%, and case fatality rates were 0% and 0.07%, respectively [11]. Based on data from Gauteng Province, South Africa, Yang et al. found that the overall risk of death from infection during the Omicron wave (0.03%; 95% CI: 0.02–0.06%) was about 30% of that during the Delta wave (0.11%; 95% CI: 0.06–0.21%) [25]. Based on data from America, Tartof et al. demonstrated that the hospitalization rates of patients with Omicron and Delta were 0.5% and 1.3%, respectively. Among patients who were positive for Omicron in the outpatient clinic for the first time, compared to patients with Delta, the hazard ratio (HR) of hospitalization and symptomatic hospitalization were 0.48 (95% CI: 0.36–0.64) and 0.47 (95% CI: 0.35–0.62), respectively. In addition, the ICU occupancy rates and case fatality rates of patients with Omicron were 0.26 (95% CI: 0.10–0.73) and 0.09 (95% CI: 0.01–0.75) times than that of Delta, respectively. Moreover, compared to patients with Delta, the length of hospitalization in patients with Omicron decreased by 3.4 days (95% CI: 2.8–4.1), and the rate of change was 69.6% (95% CI: 64.0–74.5%) [8]. In fact, compared with other variants, studies have shown that Omicron infected lung cells less easily and its replication efficiency in lung parenchyma was also lower than other variants, which may explain the reduced severity in patients with Omicron [26,27]. Of note, it was found that the proportion of asymptomatic patients with Omicron was higher than patients with other VOCs with or without previous infection caused by SARS-CoV-2 [17]. In addition, through meta-analysis, Shang found that the pooled percentage of asymptomatic infections was 32.40% among Omicron-positive individuals and that vaccinated patients had a higher proportion of asymptomatic infection [28].

## 3. Status Quo of Breakthrough Infection Caused by the Omicron Variant

According to the US Centers for Disease Control and Prevention (CDC), a vaccine breakthrough infection is defined as the detection of SARS-CoV-2 RNA or antigen in a respiratory specimen collected from a person ≥ 14 days after the receipt of all recommended doses of an FDA-authorized COVID-19 vaccine [29]. With the further enhanced transmissibility and immune escape ability of the Omicron variant, breakthrough infection occurs continuously after vaccination(Table 1). Earlier, an outbreak of Omicron occurred at a party in Oslo, Norway, on 26 November 2021, and 81 of the 110 (74%) suffered Omicron breakthrough infection [30]. A genome sequencing study of SARS-CoV-2 in the Houston Methodist healthcare system identified that 2497 (55.9%) of 4468 symptomatic patients with Omicron from late November, 2021, to 5 January 2022, met the definition of a vaccine breakthrough infection and that the proportion of Omicron breakthrough infection is significantly higher than that of Delta [31]. On 31 December 2021, 58 (89.23%) of the 65 reported infections caused by Omicron had received all recommended doses of vaccination in Guangzhou, China [32]. Based on a multicenter study in 11 general hospitals in Israel, Cohen found that, as of 31 January 2022, 4802 (20%) of the 24,280 health care workers who received boosters and 368 (7%) of the 5331 health care workers who received the fourth dose of vaccine suffered a breakthrough infection during the peak of Omicron infection [33]. Moreover, based on data from Henan, China, in January 2022, Tang reported that the breakthrough infection rate was 17.8% in the Omicron chain [34]. Nowadays, the number of persons vaccinated with at least one dose per 100 population is approximately 89 in China, which is one of the countries with the highest vaccination rate in the world, but it is experiencing a surge in breakthrough infections caused by Omicron [1].

Studies have shown that the effectiveness of all types of SARS-CoV-2 vaccines decreased to some extent in relation to the Omicron variant and that the Omicron variant has shown lower neutralizing sensitivity than other VOCs to immune sera elicited by boosters [9,35,36,37,38], even if the booster and the fourth dose can reduce the incidence of breakthrough infection and the risk of severe infection [39,40,41,42]. This indicates that some measures are needed, such as developing a second-generation vaccine, in order to prevent and control the pandemic.

## 4. Mechanism of Breakthrough Infection Caused by the Omicron Variant

As the seventh wave of COVID-19 being experienced in many regions of the world is mainly caused by Omicron subvariants BA.4 and BA.5 (referred to as BA.4/5) [43,44,45], this article mainly focuses on the BA.4/5 subvariants.

It was found that SARS-CoV-2 consists of four structural proteins, namely, the spike protein (S protein), nucleocapsid protein (N protein), membrane protein (M protein) and envelope protein (E protein) [46]. The S protein plays a critical role in the biological characteristics of the Omicron variant [10]. The S protein is a transmembrane glycoprotein located on the surface of the SARS-CoV-2 virus, which exists in the form of homotrimers and can specifically bind to angiotensin-converting enzyme 2 (ACE2) on the surface of host cells, resulting in invasion into host cells and the formation of viral syncytia mediated by type II transmembrane serine protease (TMPRSS2) [47,48]. Moreover, the S protein is post-translationally cleaved by mammalian furin into two subunits: S1 and S2. The S1 subunit [49,50], as an important target of antiviral drugs and antibodies, largely consists of the N-terminal domain (NTD) and the receptor-binding domain (RBD), which is the most variable part and is responsible for binding to the ACE2 of host cells, such as type II pneumocytes, cardiomyocytes, renal endothelium and stratified epithelium [51,52,53,54,55]. The S2 subunit, largely composed of the fusion peptide (FP), heptapeptide repeat sequence 1 (HR1), heptapeptide repeat sequence 2 (HR2), TM domain and cytoplasm domain, can be decomposed under the mediation of TMPRSS2 on the surface of host cells, promoting viral fusion, which not only accelerates the entry of RNA into host cells, but also exacerbates a large number of viral syncytia, resulting in more severe lung injury [56,57,58].

Most mAbs and vaccines currently used work against the S protein; thus, mutations in BA.4/5 S protein may significantly enhance the ability to evade vaccines [10]. It was found that the S protein sequence of BA.4/5 is the same with 31 mutations, and it is very similar to BA.2 [59]. Compared with BA.2, BA.4/5 deletes 69 and 70 residues (Del69-70), and two substitutions occur in the RBD (L452R and F486V). Moreover, BA.4/5 restored the Q493R of BA.2 to Q493 in original virus(Table 2). Hence, the S protein mutations of BA.4/5 are: T19I, Del24-26, A27S, Del69-70, G142D, V213G, G339D, S371F, S373P, S375F, T376A, D405N, R408S, K417N, N440K, S477N, T478K, E484A, Q498R, N501Y, Y505H, D614G, H655Y, N679K, P681H, N764K, D796Y, Q954H, N969K, L452R and F486V.

Among them, G339D, S373P, S375F, N440K, T478K and N501Y mutations can enhance viral binding to ACE2, resulting in enhanced viral infectivity and transmissibility [60,61,62,63]. G339D, K417N, T478K, E484A, N501Y and Y505H may decrease many S protein-directed antibodies, resulting in the enhanced ability of the virus to escape the immune system and the enhanced possibility of vaccine breakthrough infections [10,62,64]. Chen et al. found that the infectivity of Omicron with N440K, T478K and N501Y mutations was stronger than that of any previously reported variants [10]. Moreover, there are triple mutations (H655Y + N679K + P681H) near the cleavage site of furin in BA.4/5. It was found that these mutations near the cleavage site of furin enhance viral fusion ability, resulting in enhanced replication and transmissibility [65,66]. Finally, two mutations in the RBD (L452R and F486V) are of great interest in immune escape. L452R is a chemically radical change and one of two mutations in the Delta RBD. Previously, L452R in Delta was shown to enhance viral binding to ACE2 [67] and reduce memory B cell levels in individuals vaccinated, shortening the duration of memory immunity [68] and producing resistance to antibodies [69]. In addition, F486V in Omicron is also a mutation site for escaping several mAbs [70]. Moreover, both residues 452 and 486 lie close to the edge of the ACE2 interaction surface; thus, they have the potential to regulate affinity to ACE2 and neutralizing activity in serum after natural infection and vaccination [59]. Thus, L452R and F486V may enable BA.4/5 to acquire higher immune escape ability. Fortunately, the reversal of 493 may reduce escape from early viral response [59]. Properties of mutations are shown in Table 3.

Breakthrough infections occur when SARS-CoV-2 evolves, which can partially or completely escape the protective effectiveness provided by the current vaccines. Obviously, a large number of mutations in the BA.4/5 S protein pose a threat to the body’s humoral immunity, resulting in the weakening of the neutralizing activity of most antibodies induced by vaccines. Hence, breakthrough infections continue to spread, which has become the biggest threat to the current COVID-19 pandemic worldwide.

## 5. Coping Measures

Facing SARS-CoV-2, there is a global consensus to establish herd immunity, develop new vaccines and develop new therapies. We provide some potentially useful measures to help to contain the pandemic. Firstly, the establishment of herd immunity is important in combating SARS-CoV-2 and is also the only effective way to prevent COVID-19 [72,73]. However, the current vaccine distribution is unbalanced. Only a few people have received at least one dose in some low-income countries, while the fully vaccinated rate has reached 60–90% in some developed countries [74]. In addition, as the anti-vaccine campaigns grow stronger, hesitancy related to SARS-CoV-2 vaccines has been maintained at a high level among both citizen and health care workers [75]. Therefore, countries around the world should actively respond to the appeal by the WHO to prevent variants from spreading further by adjusting the distribution of vaccines, completing vaccination strategies and establishing herd immunity as soon as possible. Secondly, based on the current vaccines, countries should accelerate the development of the second-generation vaccine that is prophylactic and better able to respond to new variants. In most patients with breakthrough infection, the virus has been detected in the upper respiratory tract only, with no or mild symptoms of pulmonary infection. Some scholars suggest that nasal vaccines may more effectively mediate prophylaxis in the respiratory tract as they can produce memory B and T cells in the respiratory mucosa, based on studies using nasal vaccines that appeared to be superior to an intramuscular injection in controlling the respiratory viral load [76,77]. Hence, the nasal mucosal vaccine is expected to become an effective new strategy to prevent breakthrough infection. Moreover, cytotoxic T cells can disinfect by killing virus-infected cells. Studies have confirmed that specific T-cell immunity targeting SARS-CoV-2 proteins plays an important role in viral clearance and prevention [78,79]. Some scholars found that neutralizing antibody levels significantly decreased in recovered patients > 3 months after infection, but T-lymphocyte immunity persisted after at least 10 months [80,81,82] and is expected to be maintained for many years [83]. This is similar to previous SARS-CoV studies [84]. Hence, T-lymphocyte immunity could be taken into account when developing the next-generation vaccine. Finally, despite the impact of resistance caused by Omicron mutations on existing treatment [64], Tuekprakhon [59] found that Cilgavimab (AZD1061) achieved an IC_50_ =0.019 μg/mL and 0.015 μg/mL in relation to BA.4 and BA.5 through pseudoviral neutralization experiments, respectively, indicating that it is still a therapeutic option for BA.4/BA.5 [5,59]. Moreover, nanobodies have promising applications against respiratory viruses due to their powerful physicochemical properties that allow for inhalation delivery [85]. Lu et al. obtained nanobodies targeting the S protein (Nb91-hFc) and targeting the RBD (Nb3-hFc) by screening a naïve nanobody library. Pseudoviral neutralization experiments have showed that the IC_50_ of Nb91-hFc and Nb3-hFc were 2.65 μg/mL and 1.79 μg/mL, respectively [86]. In addition, it was demonstrated that multivalent nanobodies were more efficient against virus compared with monovalent antibodies [87,88]. Li et al. obtained the nanobody MR3 from a camelid nanobody library, which had high neutralizing activity (IC_50_ = 0.40 μg/mL) against SARS-CoV-2 pseudovirus, and the neutralizing activity could be further enhanced (IC_50_ = 12 ng/mL) by converting the monovalent nanobody into a multivalent or multi-specific nanobody after polymerization by linking sequences [89]. Hence, nanobodies have great development potential for the treatment of COVID-19.

## 6. Conclusions

Studies on the mechanism of breakthrough infection caused by the Omicron variant mainly focus on the S protein at present. Hence, this article mainly includes studies related to the S protein, but other proteins may also play an important role in breakthrough infection. Moreover, part of the references included in the status quo section did not specify the vaccine type, which is one of the limitations of this article.

In conclusion, breakthrough infection caused by the Omicron variant continues to occur, and the conformational change in the S protein plays an important role. Hence, knowing further information about breakthrough infection caused by the Omicron variant and taking some potentially helpful measures are of great significance in preventing and controlling the pandemic more effectively.

## Figures and Tables

**Table 1:** Breakthrough infection caused by the Omicron variant in the literature.

References	Type of Study	Period of Study	Country	Sample Size	Diagnosis Method	Proportion of Breakthrough Infection	Vaccine Type
Brandal [30]	Cohort study	2021.11.26~2021.12.3	Oslo, Norway	110	PCR variant screening; whole genome sequencing	71.8%	BNT162b2 (50.1%); mRNA-1273 (21.0%)
Christensen [31]	Case-control study	2021.11.27~2022.1.5	Houston, America	4468	S-gene target-failure assay	55.9%	BNT162b2(73%); mRNA-1273 (22%); JNJ-78436735 (5%)
Hu [32]	Case-control study	2021.12.13~2021.12.31	Guangzhou, China	65	second-generation sequencing	89.2%	─
Cohen [33]	Cohort study	2022.1	Israel	29,611 (the booster: 24,280; the fourth dose: 5331)	polymerase chain reaction test	the booster: 20.0%; the fourth dose: 5.0%	BNT162b2 (100%)
Tang [34]	Cohort study	2022.1.2~2022.1.23	Henan, China	2208	─	17.8%	BBIBP-CorV and CoronaVac (91.8%); ZF2001 (7.5%); Ad5-nCoV (0.7%)

**Table 2:** Mutations in the spike protein of Omicron subvariants: BA.2 and BA.4/5.

Subvariants	Mutations
BA.2	T19I,Del24-26,A27S, G142D,V213G,G339D,S371F,S373P,S375F,T376A,
BA.4/5	T19I,Del24-26,A27S,Del69-70,G142D,V213G,G339D,S371F,S373P,S375F,T376A,
BA.2	D405N,R408S,K417N,N440K, S477N,T478K,E484A, Q493R,Q498R,
BA.4/5	D405N,R408S,K417N,N440K,L452R,S477N,T478K,E484A,F486V, Q498R,
BA.2	N501Y,Y505H,D614G,H655Y,N679K,P681H,N764K,D796Y,Q954H,N969K
BA.4/5	N501Y,Y505H,D614G,H655Y,N679K,P681H,N764K,D796Y,Q954H,N969K

**Table 3:** Properties of mutations in the literature.

References	Mutations	Properties
Vo, 2022 [61]	S373P,S375F, T478K,E484A	Enhance viral binding to ACE2
Kumar, 2022 [63]	G339D,N44OK,S373P,S375F,E484A	Enhance viral binding to ACE2
Queiros-Reis, 2021 [62]	T478K,N501Y	Enhance viral binding to ACE2 and decrease S protein-directed Abs
Chen, 2022 [10]	G339D,K417N,E484A,Y505H	Decrease S protein-directed Abs
Wang, 2022 [71]	K417N,T478K,E484A	Decrease S protein-directed Abs
Chen, 2022 [10]	N440K, T478K, N501Y	Enhance viral infectivity
Dhawan, 2022 [65] El-Shabasy, 2022 [66]	H655Y + N679K + P681H	Enhance viral fusion ability
Sapkal, 2021 [67] Haralambieva, 2022 [68] Deng, 2021 [69]	L452R	Enhance viral binding to ACE2 and decrease memory immunity and resist Abs
Gobeil, 2021 [70]	F486V	Escaping mAbs

## Data Availability

Not applicable.
